# Improved detection of global copy number variation using high density, non-polymorphic oligonucleotide probes

**DOI:** 10.1186/1471-2156-9-27

**Published:** 2008-03-28

**Authors:** Fan Shen, Jing Huang, Karen R Fitch, Vivi B Truong, Andrew Kirby, Wenwei Chen, Jane Zhang, Guoying Liu, Steven A McCarroll, Keith W Jones, Michael H Shapero

**Affiliations:** 1 Affymetrix, Inc. 3420 Central Expressway; Santa Clara, CA 95051, USA; 2 Center for Human Genetic Research, Massachusetts General Hospital, Boston, MA 02114, USA; 3 Program in Medical and Population Genetics, Broad Institute of MIT and Harvard, Cambridge, Massachusetts 02142, USA

## Abstract

**Background:**

DNA sequence diversity within the human genome may be more greatly affected by copy number variations (CNVs) than single nucleotide polymorphisms (SNPs). Although the importance of CNVs in genome wide association studies (GWAS) is becoming widely accepted, the optimal methods for identifying these variants are still under evaluation. We have previously reported a comprehensive view of CNVs in the HapMap DNA collection using high density 500 K EA (Early Access) SNP genotyping arrays which revealed greater than 1,000 CNVs ranging in size from 1 kb to over 3 Mb. Although the arrays used most commonly for GWAS predominantly interrogate SNPs, CNV identification and detection does not necessarily require the use of DNA probes centered on polymorphic nucleotides and may even be hindered by the dependence on a successful SNP genotyping assay.

**Results:**

In this study, we have designed and evaluated a high density array predicated on the use of non-polymorphic oligonucleotide probes for CNV detection. This approach effectively uncouples copy number detection from SNP genotyping and thus has the potential to significantly improve probe coverage for genome-wide CNV identification. This array, in conjunction with PCR-based, complexity-reduced DNA target, queries over 1.3 M independent NspI restriction enzyme fragments in the 200 bp to 1100 bp size range, which is a several fold increase in marker density as compared to the 500 K EA array. In addition, a novel algorithm was developed and validated to extract CNV regions and boundaries.

**Conclusion:**

Using a well-characterized pair of DNA samples, close to 200 CNVs were identified, of which nearly 50% appear novel yet were independently validated using quantitative PCR. The results indicate that non-polymorphic probes provide a robust approach for CNV identification, and the increasing precision of CNV boundary delineation should allow a more complete analysis of their genomic organization.

## Background

With the completion of the human genome sequence, it is generally accepted that any two individuals are ~99.9% identical at the nucleotide level, and that the presence of single nucleotide polymorphisms (SNPs) in the genome are the major contributor to genetic diversity among humans [1]. In part due to the accuracy and ease in which they can be scored, along with their stability and abundance in the genome, SNPs have become the marker of choice for whole genome association studies that use linkage disequilibrium (LD) mapping to identify genes involved in complex diseases [2,3]. Over the last several decades, it has also been accepted that there can be DNA copy number changes that occur among individuals, albeit in the context of limited and specific loci within the genome. These changes can span a spectrum from, for example, an extra copy of an entire chromosome (trisomy 21) in Down's syndrome to sub-chromosomal deletions responsible for genetic traits such as color blindness and α and β thalassemias [4]. However, this paradigm of genetic variation underwent a major revision in 2004 with the identification of genome-wide copy number variants that occur among phenotypically normal individuals [5,6]. Since these initial reports, a large number of studies have described the wide spread and global distribution of CNVs in the genome [7-17]. As the cataloguing of CNVs in the genome continues, new studies are also aimed at understanding their function in normal cellular processes such as drug metabolism [18,19] and gene expression [20], in human disease susceptibility [21-23] and developmental disorders [24], and in the natural selection process [25]. Lastly, the role of CNVs in genomic disorders further underscores how profoundly gene function can be adversely affected in a multitude of ways that can lead to disease [26-29]. Recent estimates of the contribution of CNVs to total nucleotide diversity per genome range from 9 to 30 Mb and thus exceeds the ~3 Mb estimated to be due to SNPs [7,9,30]. In fact, a recent comparison of the genome sequence of an individual human with the NCBI human reference assembly suggested that DNA copy number variable regions contribute ~10 Mb to sequence heterogeneity [31]. These results underlie the growing appreciation for and understanding of the need to account for CNVs in genome wide association studies. Although some common CNVs are in LD with SNPs and can therefore be assayed indirectly through SNP genotyping, a significant fraction of CNVs (particularly those in duplication-rich regions of the genome) are not well-captured by available SNP marker sets [7,12,14,32]. Furthermore, even taggable CNVs need to be accurately typed before appropriate markers can be identified. Thus there is still an on-going need to develop molecular methods capable of direct and accurate detection of CNVs in order for this new class of polymorphisms to be effectively incorporated into genome wide LD mapping of genes involved in human disease [33].

There is a wide range of structural variation that can occur in the genome that includes deletions, insertions, duplications, and inversions, and these can range from 1–500 bp (fine-scale), 500 bp–100 kb (intermediate-scale), and >100 kb (large-scale) in size. Although there are many different molecular cytogenetic techniques that can be used to assess variants when one or several specific targeted loci are under investigation [26,34,35], there are only a limited number of approaches that provide genome-wide characterization, namely direct sequencing approaches such as fosmid paired-end sequencing [15] or Paired-End Mapping (PEM) [30] and array-based methods. Array-based methods that have been applied to CNV identification include the use of BAC clones [5,7-9] and both long [6,36] and short oligonucleotide probes [7,12,37]. We have reported in 2006 on a comprehensive analysis of CNVs in the HapMap DNA collection using two complementary platforms, namely BAC-array CGH and 500 K EA high-density genotyping array. While these two approaches often identified the same CNVs, there were differences in the types of CNVs unique to each approach. For example, while the 500 K EA array tended to identify smaller CNVs along with higher border resolution, the BAC array CGH approach was able to interrogate regions of the genome that are not easily amenable to SNP genotyping due to the presence of low copy repeat structures (segmental duplications). As a means to uncouple the requirement of SNP genotypes from CNV identification, we have designed and evaluated an array that uses non-polymorphic 25-mer probes in combination with a PCR-based, reduced complexity DNA target. This array has been used for high resolution analysis of DNA deletions in Gorlin syndrome samples [38], and in this report we show using a well-characterized pair of DNA samples, in conjunction with a novel CNV detection algorithm, that nearly 200 CNVs are identified, of which over 120 had not previously been described in this specific sample pair. All novel CNVs were evaluated using an independent QPCR based method, and the overall results show a verification rate of nearly 85%. Thus, DNA probes designed to sites in the genome that do not contain SNPs are effective for CNV identification, and when combined with probes used for SNP genotyping, provide a potentially powerful approach for the integration of CNVs and SNPs into genome wide association studies.

## Results

Whole genome sampling analysis (WGSA) uses single primer PCR in combination with adapter-ligated, restriction enzyme-digested genomic DNA as template to selectively and reproducibly amplify genomic fractions [39]. Based on *in silico *NspI restriction enzyme digestion of the human reference genome (Build 35), over 1.33 million independent fragments are predicted in the 200 bp to 1100 bp size range. The 500 K EA array, which was previously used for genome-wide CNV detection, uses both NspI and StyI PCR representations on two individual arrays. In this configuration, the NspI WGSA target interrogates ~250 K SNPs which in general each reside on a unique restriction fragment. Thus only ~20% (0.25 M/1.3 M) of the *in silico *predicted NspI fragments are estimated to be represented on the 500 K EA array in the form of probes querying SNPs. Since the NspI PCR target has an estimated complexity of 550 Mb, it could potentially serve as a means to interrogate a significant fraction of the genome provided that two key criteria are met, namely, that these sequences can be reliably amplified by PCR during WGSA and that probes for all fragments are represented on the array and function in a specific manner in DNA hybridization. To this end, a new array was designed using non-polymorphic probes (referred to as the Nsp copy number (CN) array) for the goal of CNV detection.

**Figure 1:**
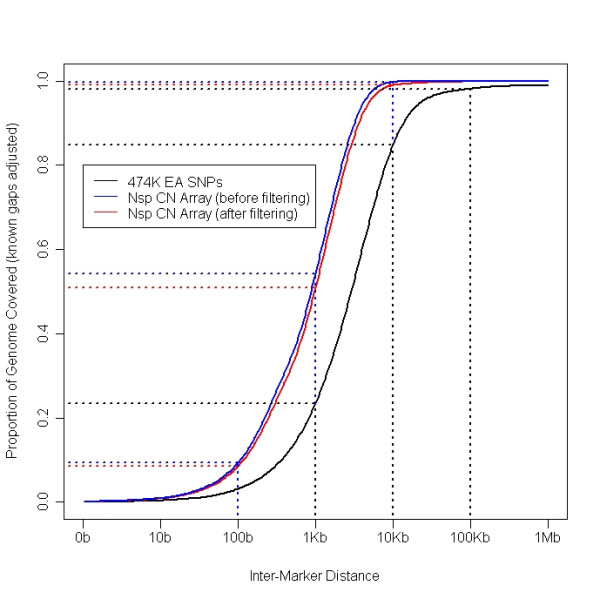
**Genome coverage of the Nsp CN array before and after probe filtering compared with 500 K EA arrays**. The X-axis is the distance between any given point in the gap-adjusted genome and the next closest marker. The curve shows the proportion of the genome where the closest marker is less than a certain distance. For example, for the after probe filtering Nsp CN array markers, 99.0% of the genome is less than 10 kb away from a Nsp fragment marker (compared to 99.8% for the before probe filtering Nsp CN array markers) while for the 500 K EA selected SNPs, only 84.9% of the genome has a SNP within 10 kb.

The Nsp CN array contains eight to ten independent, non-polymorphic probes per restriction fragment which were selected based on intrinsic criteria (see Methods). Globally, these arrays, in combination with NspI WGSA target only, result in an increase in probe coverage when compared to the 500 K EA genotyping arrays which used both NspI and StyI WGSA fractions (Figure 1). The median inter-marker distance for the Nsp CN arrays is 776 bp, compared to 2709 bp for 500 K EA probes [37]. As expected, genome coverage is improved. For example, at an inter-marker distance of 2.5 Kb, the 500 K EA array covers ~46% of the genome whereas coverage increases to over 84% with the Nsp CN array. Because the selection of probe sequences is no longer constrained to SNPs, this array design also has improved coverage in regions likely to contain CNVs, such as segmental duplications [8]. For example, while only 25.7% of segmental duplications contain at least one SNP found on the 500 K EA array, 90.3% of segmental duplications are represented by probes from at least one restriction fragment on the Nsp CN array before probe filtering (Table 1).

**Table 1:** Coverage of segmental duplication regions by 500 K EA and Nsp CN arrays.

	**500 K EA**		**Nsp CN array**									
			Before probe filtering		After probe filtering	After local-correction filtering		After probe filtering	After local-correction filtering		After probe filtering	After local-correction filtering

					**Data set 1**			**Data set 2**			**Data set 3**	

At least one marker	25.7%		90.3%		74.1%	73.5%		74.3%	73.8%		74.0%	73.0%
At least two markers	13.4%		85.2%		61.7%	60.5%		61.8%	60.7%		61.6%	60.3%
At least three markers	7.7%		78.1%		50.4%	49.2%		50.7%	49.5%		50.2%	49.1%
At least four markers	5%		69.7%		40.7%	39.1%		41.0%	39.3%		40.7%	39.3%

Note: Each data set represents a replicate of 1X–5X samples. For 500 K EA, marker refers to SNPs; For Nsp CN array, markers refer to Nsp fragments.

Segmental duplication data source [80]

### Assay and array performance

Although the human reference genome is commonly used to predict outcomes of *in silico *restriction enzyme digestions, the precise relationship between all expected fragments, regardless of whether they contain a SNP or not, and the WGSA target output has not been systematically evaluated [40,41]. The Nsp CN array, which contains multiple independent probes per fragment, was used to evaluate how well each fragment is represented by the WGSA assay. For this purpose, the difference was estimated between probe-specific background (using a pooled panel of 'antigenomic' probes that are not present in the human genome and which vary in GC content in a similar manner to the perfect match probes [42]), and the target-dependent probe signal using a set of five genomic DNA samples that contain different numbers of X chromosomes (designated as the 1X to 5X sample set). Using a probe sequence-specific background model (see Methods), >97% of all probes show an intensity that is higher than background in each individual sample and > 94% of all probes are detected above background when all 5 samples are evaluated together as a group (Table 2). Although this metric does not measure the specificity of the signal per se but rather whether the signal is real or not in terms of being above background level, it does suggest that nearly all predicted restriction fragments are actually represented in the PCR target at a concentration sufficient for detection by hybridization. The small remaining set of non-responsive fragments could result from problems with restriction enzyme digestion, PCR amplification, hybridization, or sequence differences between the human genome reference sequence and the genomes of the samples being tested.

**Table 2:** Estimation of number of probes that respond to target and display an intensity above the background

	**Probes above background in each sample**		**Probes and fragments above background in 5/5 samples**			
	**Probe count**	**Percentage**	**Probes # (%)**		**Fragment # (%)**	

	**data set 1**					

**Sample1**	12,017,471	97.47%	11,786,082	(95.59%)	1,329,822	(99.96%)
**Sample2**	12,025,953	97.54%				
**Sample3**	12,075,266	97.94%				
**Sample4**	12,092,454	98.08%				
**Sample5**	12,080,046	97.97%				

	**data set 2**					

**Sample1**	11,980,266	97.17%	11,697,525	(94.87%)	1,329,806	(99.96%)
**Sample2**	12,053,875	97.76%				
**Sample3**	12,056,015	97.78%				
**Sample4**	12,039,968	97.65%				
**Sample5**	11,981,189	97.17%				

	**data set 3**					

**Sample1**	11,965,896	97.05%	11,687,506	(94.79%)	1,329,818	(99.96%)
**Sample2**	12,061,150	97.82%				
**Sample3**	12,027,025	97.54%				
**Sample4**	12,060,619	97.82%				
**Sample5**	12,040,767	97.66%				

Note: Each data set represents a replicate of 1X–5X samples.

The probes present on the Nsp CN array have not been experimentally selected *a priori *for high performance with regard to detection of DNA copy number changes. In order to test if these probes are sensitive to changes in target dosage, the 1X to 5X DNA samples were used in WGSA and target was hybridized to the arrays for the purpose of X chromosome probe evaluation. Using all probes present on the X chromosome, a clear increase in signal was seen with increasing X chromosome dosage (Additional File 1). These results confirm that probes on the Nsp CN array display a dose response for the X chromosome. The use of these DNA samples also allows assessment of individual probe-specific dose response metrics (i.e. regression slope and linear correlation coefficient). For example, under ideal theoretical conditions, a single probe that maps to only one site on the X chromosome, when evaluated with the 1X to 5X sample set, would show a regression slope value of 1 when the linear regression is modeled using the log-transformed intensity as the response and the log-transformed copy number as the predictor. Similarly, a linear correlation coefficient of 1 would be expected. Thus, deviation from these ideal values provides an experimental approach to measuring each probe's ability to respond to changes in target concentration. Two examples are shown in Additional Files 2 and 3.

The impact of the number of genomic hits on probe dose response was also evaluated using the X chromosome probe intensities from the 1X–5X data set (Additional File 2). Linear correlation between log (probe intensity) and log (chrX copy number) was calculated for each of the chrX probes after grouping probes by number of perfect-match genomic hits. The Pearson's correlation coefficient of each group (Additional File 2B) dramatically decreased when the number of genomic hits was greater than two. The log (probe intensity) and log (chrX copy number) was further modeled by simple linear regression. Again, the regression coefficient (regression line slope, as shown in Additional File 2C) grouped by number of genomic matches indicated poorer performance when the probes were complementary to more than two sites in the genome. The same analyses stratifying on the number of chromosome X hits using the same set of chrX probes gave similar results (Additional File 2D–2F). Although these metrics were also smaller for probes with two-genomic matches as compared to single-match probes, the magnitude of the reduction was not as large relative to the change from two-genome matches to three or greater genomic matches. More importantly, since many CNVs are associated with segmental duplication regions, there is an increased likelihood for probes in CNV regions to have two genome hits. Thus, probes with two genome hits were not omitted in order to allow interrogation of segmental duplication regions (Table 1), while probes that have more than two genomic hits were removed as described in Methods.

Several probe filtering steps were implemented in addition to the probe filtering described above for genomic hits in order to remove adversely performing probes (see Methods). These additional procedures included filtering based on probe GC content, restriction fragment length and GC content, NspI restriction site characteristics, hybridization signal intensities lower than background, hybridization signals that are too bright, and probe sets comprised of single probes. Following the probe filtering steps, sequence specific standardization was performed and the probes from each restriction fragment were summarized as described in Methods. At the completion of all filtering steps, ~77% of the initial probes and 92% of the initial restriction fragments were retained in a typical experiment, although the exact number varied dynamically for each sample set that was analyzed together (Additional File 4). Importantly, genome coverage was not significantly reduced by probe filtering (Figure 1) although coverage in segmental duplication regions with at least one marker was modestly reduced from 90% to 74% (Table 1). The overall impact of probe filtering as well as a median polish procedure (Robust Multichip Analysis (RMA)) on dose response was evaluated using the 1X–5X sample set dose response metrics. The linear correlation coefficient and the regression slope improved significantly in both cases (Additional File 5).

### Detection of copy number polymorphisms

**Figure 2:**
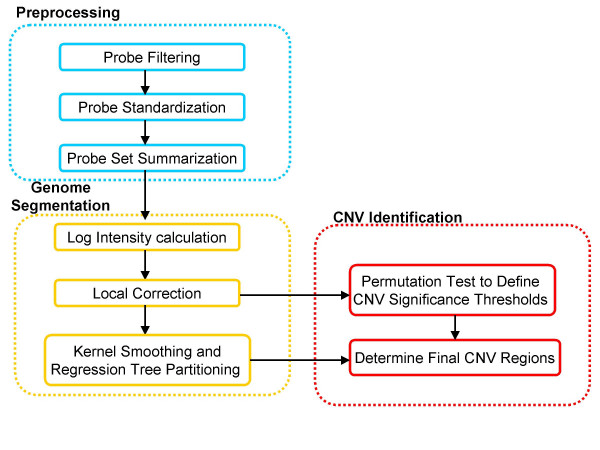
**Overview of the data analysis work flow **(see Methods for details).

To evaluate the capability of the Nsp CN array to identify CNVs, multiple independent replicates of two well characterized DNA samples (NA15510 as the test sample and NA10851 as the reference sample) that contain known copy number variations were used. Although CNVs in these two samples have previously been identified using high density oligonucleotide arrays [7,37], we hypothesized that improved probe density in regions devoid of SNPs, such as segmental duplications, should lead to the discovery of additional variants. For this purpose, a novel algorithm was developed to identify copy number variation regions. This algorithm contains three major parts as depicted in Figure 2. Intensity pre-processing includes probe filtering, standardization which takes into account probe specific metrics known to influence hybridization and signal intensity, and probe set summarization to provide a single measurement for each fragment. The genome segmentation step initially removes outlier fragments, uses kernel smoothing to improve the signal to noise ratio, and then applies a regression tree based method to divide the genome into consecutive regions. Lastly, CNV region identification is achieved by a permutation based test to define the significance threshold. The training set data for tuning various algorithm parameters (see Methods) consisted of a single replicate of NA15510 compared to NA10851. Tuned parameters were then used in subsequent analyses that included two independent test sets of NA15510 versus NA10851 as well as several HapMap trio samples.

**Figure 3:**
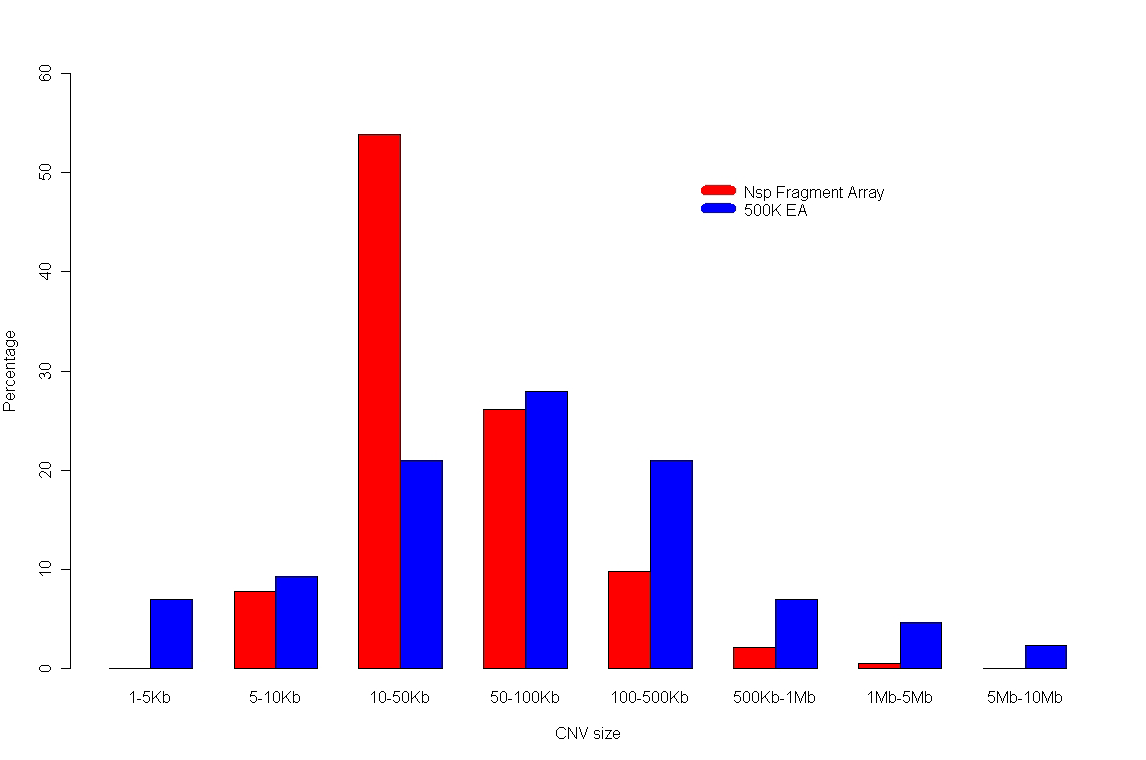
Size distribution of CNVs detected using the Nsp CN array (red bars) compared with 500 K EA (blue bars) CNVs.

Using the two independent test replicates between NA15510 and NA10851, 195 high confidence CNVs were identified in total (gains (98) and losses (97) were represented nearly equally), with 156 CNVs and 175 CNVs found in each of the two pair-wise comparisons. This represents, on average, a five fold increase over the number of CNVs identified in this same sample pair using 500 K EA arrays [37]. In total, 10,126,153 nucleotides were included in these CNV regions, representing 0.355% of the gap-adjusted genome size, and 39.5% of the CNVs overlapped with segmental duplications (Additional File 6A). The mean and median size of CNVs identified on the Nsp CN array were significantly smaller as compared to CNVs found on the 500 K EA arrays (51,930 bp and 20,780 bp versus 293,800 bp and 48,950 bp respectively), a direct result of the improved probe coverage (Figure 3). There were 121 CNVs identified in both sample sets, corresponding to a reproducibility rate of ~77% (Additional File 6). There have been several reports describing CNVs found in this specific pair of samples using multiple detection platforms such as fosmid paired-end sequencing, whole genome tile path (WGTP) BAC array CGH, and 500 K EA arrays [7,15]. The overlap of the 195 CNVs with this external data set identified 73 CNVs (37.44%) (Additional File 6), and thus these were considered to be validated based on the criteria of overlap with previously described CNVs found in these two samples. Interestingly, the average size of CNVs that overlapped with external data was 91,536 bp as compared to an average size of 28,229 bp for those CNVs that did not overlap with external data. By virtue of no overlap with the external data sets, there were 122 novel CNVs. 120 of these 122 CNVs were tested by QPCR and the results showed that 94/120 (78.3%) could be validated (Additional File 6), indicating that the majority of the novel CNVs represented real but previously unidentified structural variation between NA15510 and NA10851. Taken together, the percentage of the 195 total CNV calls that were validated (based on a combination of external data set overlap and QPCR analysis) was 86.5% and the percentage of CNV calls from each pair-wise comparison that was validated was near 89% (Additional File 6). To assess the number of false-positive CNV calls using this array and algorithm, 'self versus self' comparisons using the NA10851 reference sample were carried out. An average false discovery rate of 7.3% was determined (avg # CNV calls NA10851 vs NA10851/avg # CNV calls NA10851 vs NA15510), which is similar, although slightly lower, than the experimentally identified rate of false positive calls of 11% (100%-89%) for a test versus reference pair-wise sample comparison.

**Figure 4:**
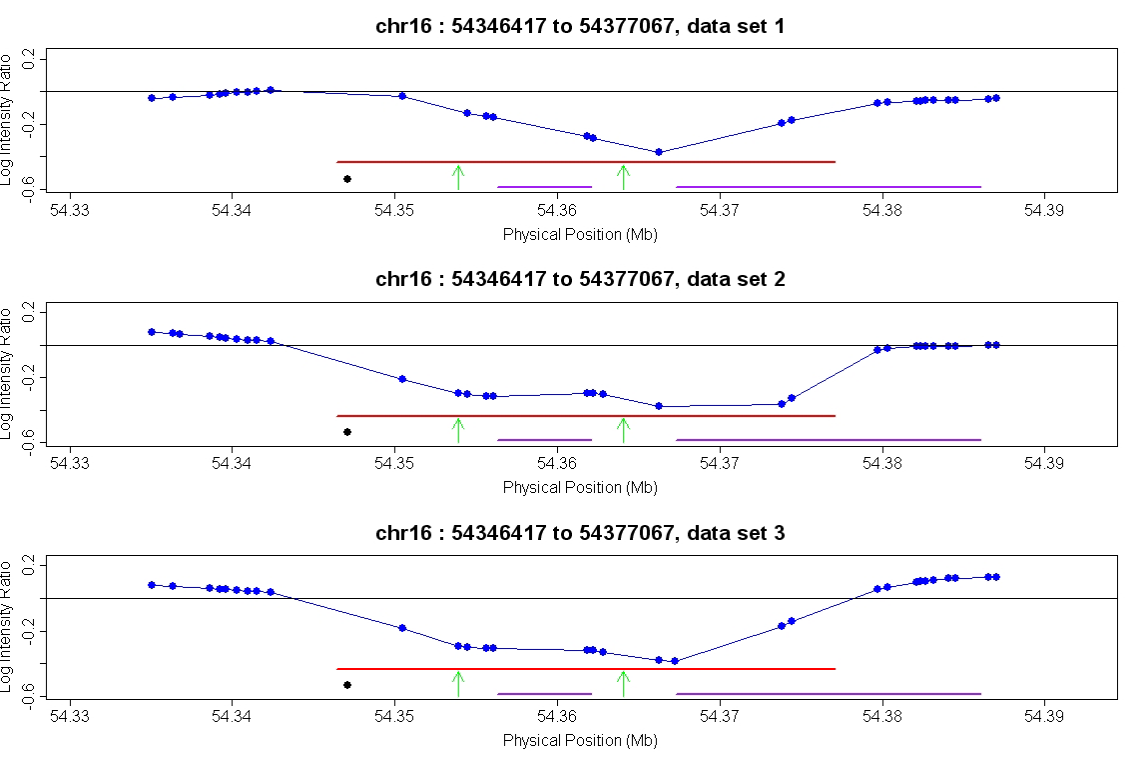
**Improved ability to detect CNVs in segmental duplication regions**. In this CNV region associated with two segmental duplications, there is one SNP probe on the edge of the region (54347071 bp on chromosome 16, represented by the black dot) on the 500 K EA array, but multiple probes present on the Nsp CN array. The three panels represent three independent replicates (one training replicate (data set 2) and two test replicates (data set 1 and data set3)) of the test sample NA15510 and the reference sample NA10851 on the Nsp CN array. The log intensity ratios are plotted on the Y axis and the genomic location on the X axis. The red horizontal line represents the CNV region identified by the Nsp CN array and algorithm, while the purple horizontal lines represent segmental duplication regions. The green arrows indicate location of primers used for QPCR verification (listed in Additional File 6).

Regions containing low copy repeats are often not detectable with SNP genotyping arrays since SNPs in these regions do not typically perform well [43]. The Nsp CN array contains non-polymorphic probes that are more likely to span duplicated regions, and thus the power to detect CNVs surrounding segmental duplications is increased. From our union list of CNVs identified from two replicates of NA15510 vs NA10851, we identified 77 CNVs (39.5%) that are associated with segmental duplications (Additional File 6), compared to 18 CNVs from a similar data set using the 500 K EA array [7]. Figure 4 illustrates a CNV associated with a segmental duplication.

**Figure 5:**
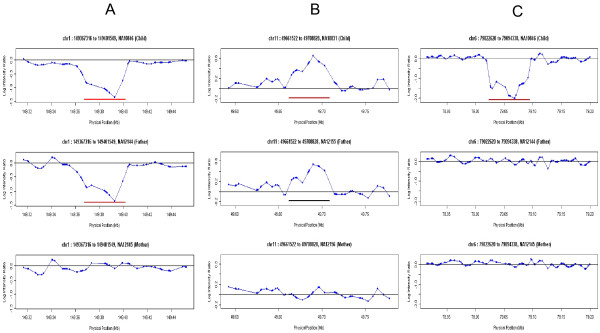
**CNV inheritance patterns in two family trios**. Although most CNVs are clearly inherited (Figure 5A) or displayed an intensity profile in one of the parents that is just below the threshold cutoff (Figure 5B), there are CNVs that appear to be de novo (Figure 5C). This could be due to complicated inheritance of a common CNV present in both parents and the reference, a false positive in the child, or a de novo event in the child. The log intensity ratios are plotted on the Y axis (the dots represent the log intensity ratio of each probe) and the genomic location on the X axis. Red horizontal lines represent CNVs identified in our study and the black horizontal line in Figure 5B represents the same region in the parent that was identified in the child sample as a CNV region. (A) Transmission of a CNV from a father (NA12144) to the child (NA10846). (B) Transmission of a CNV from a father (NA12155) to the child (NA10831). In this case, the intensity profile in this region in the father is just below the significance threshold and was not called as a CNV. However, this region displayed a strong trend as a CNV. (C) A deletion CNV identified in the child (NA10846) is not found in either of the parents (NA12144 and NA12145).

CNVs have previously been shown to be largely heritable [7,8,14]. As such, the performance of the CNV detection assay and algorithm was assessed by evaluating Mendelian inheritance (MI) of CNVs in two trios that are part of the HapMap collection of DNA samples of Caucasian (CEU) descent (Figure 5). The 6 samples that comprise the two trio sets were each compared to the reference sample (NA10851). Thus, all CNVs derived from these comparisons are a composite of copy number variation in the test sample as well as the reference sample. This analysis showed that 95.1% of CNVs (157/165) identified in the 2 children of these trios were also found in at least one of the parents. This includes 113 CNVs that were called by the algorithm in both the child and parent and are classified as inherited (Figure 5A) as well as 44 CNVs with signal intensities in one of the parents that were just below the significance threshold cutoff and are classified as "display MI trend" (Figure 5B, Additional File 6E). The remaining CNVs could represent detection errors (false positive CNVs in the child or false negative CNVs in either parent), a "de novo" event in the child, a cell line artifact in the child's sample [7], or an inherited CNV that has a more complicated inheritance pattern (Figure 5C). To evaluate these possibilities, all eight non-inherited CNVs were evaluated for overlap with previously released data sets that used the same samples [7,11,14,32] and were also experimentally evaluated using QPCR (Additional File 6D). This analysis showed that 4 of the 8 non-inherited CNVs were truly present in the child's sample, but were not detected in the parent's samples.

A comparison of the four validated "de novo" CNVs with CNVs that have previously been described in the literature for these samples reveals that one of these four can be categorized as a CNV with a complex inheritance pattern and a second CNV can be categorized as a putative cell line artifact. In the case of the trio which includes the child DNA sample NA10846, a "de novo" CNV from 79,022,620 bp to 79,094,338 bp on chromosome 6 was validated using several QPCR primer pairs targeting different regions of the CNV (Figure 5C). In a previous study [7], this common CNV region was identified as a deletion in both parent samples (NA12144 and NA12145) as well as the reference sample (NA10851), and was found to be a homozygous deletion in the child (NA10846). Because the reference sample and the two parents contain the same CNV allele, the presence of the deletion in the parents was masked in our study. Thus, this is an example where an apparently "de novo" or non-inherited CNV appears to follow simple Mendelian inheritance but is missed due to the configurations of genotypes in the tested samples relative to the reference sample. In another example, for the case of the trio NA10831-NA12145-NA12146, a "de novo" CNV was validated between 84,014,256 bp and 84,037,846 bp on chromosome 7, but only in a specific lot number of the DNA sample corresponding to the child (Additional File 6). In previous work, this region was identified as a deletion in the child sample (NA10831), but not in the parent samples (NA12145 and NA12146) and was thus flagged as a potential cell line artifact [7].

### High resolution breakpoint determination for CNVs

**Figure 6:**
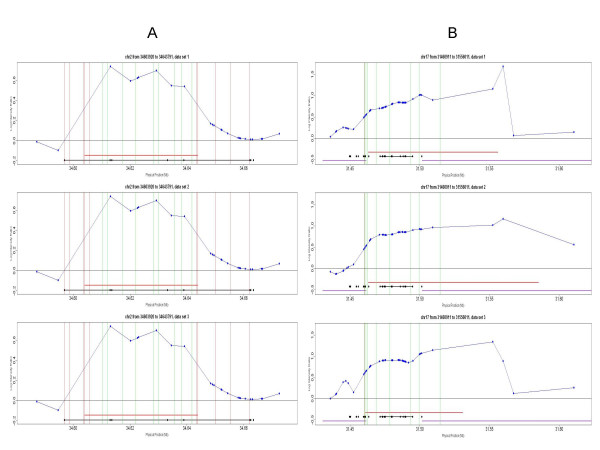
**Improved boundary delineation with Nsp CN arrays compared to 500 K EA**. The CNV in these examples were identified by both the 500 K EA platform (black lines) as well as the Nsp CN array (red lines). The three panels represent three independent replicates of the test sample NA15510 and the reference sample NA10851 on the Nsp CN array (data set 1 and data set 3 are test data sets and data set 2 is used as training set). The blue lines represent the log intensity ratios, with the dots indicating the location of each probe from the Nsp CN array. Colored vertical lines indicate different primer pairs, with green indicating a confirmed copy number change, and red indicating no detectable copy number change. The black dots on the black horizontal line represent SNP markers tiled on the 500 K EA arrays. A) This CNV was identified as a 40 kb insertion using the Nsp CN array, and a 65 kb insertion using the 500 K EA arrays. The primer pairs, ordered from left to right on the figure, are named 1 to 19 in Additional File 6C. B) This CNV was identified as a 95 kb insertion using the Nsp CN array and a 23 kb insertion using 500 K EA. In addition, the CNV is flanked by segmental duplications (purple lines). Primers 1 through 9 are numbered from left to right in Additional file 6C.

For the Nsp CN array, the CNV border was defined as the middle point between the outer most fragment present in a region showing significance and the nearest fragment located outside of the significant region. For this reason, the reported border for a CNV region is an approximation of the true border, which should lie somewhere between these two points. The accuracy of the array and algorithm to delineate CNV boundaries was evaluated by experimental testing of 2 CNV regions that were identified by both the Nsp CN array as well as the 500 K EA platform (Additional File 6C). The first CNV tested was identified as a 40 kb insertion on chromosome 2 by the Nsp CN array and a 65 kb insertion by 500 K EA (Figure 6A). QPCR primers were designed to the regions immediately adjacent to the borders defined by the Nsp CN array, internal to the defined borders, and to regions that differed between the two platforms. The results show that the borders defined by the Nsp CN array and algorithm were highly accurate and limited only by the density of markers in the region (Figure 6). A comparison of the borders reported by the Nsp CN array and the borders reported by the 500 K EA array with the experimental QPCR results shows that the higher density of markers in the Nsp CN array is beneficial in the identification of the true border of a CNV region.

A second example was tested which was defined as a larger CNV by the Nsp CN array (95 kb insertion on chromosome 17) compared to 500 K EA (23 kb insertion on chromosome 17). The primary reason for the smaller size on the 500 K EA platform was the lack of SNP probes in the segmental duplications that are associated with this CNV (Figure 6B). Again, the Nsp CN array borders were found to be more accurate (Additional File 6). It should be noted that although this CNV is clearly larger than 23 kb, the precise borders were difficult to establish due to the presence of segmental duplications within and flanking the region (Figure 6B).

## Discussion and Conclusion

The routine testing of CNVs during genome wide association studies has been widely proposed yet has not been fully realized to the same extent as SNP genotyping [44-46]. This goal is hindered in part by the fact that accurate and sensitive detection of CNVs that span varying numbers of nucleotides poses greater technical challenges than the genotype determination of a bi-allelic single nucleotide polymorphism. In addition, although SNPs can reliably be identified by many different molecular assays which all result in a common output (homozygous or heterozygous genotype call), CNV outputs can vary widely depending on the specific technical platform, calling algorithm, and reference DNA sample that is used [47,48].

The ability to accurately assess common copy number variation requires the development of novel high throughput technologies as well as the algorithms to extract and process the appropriate information. Here we describe a high density oligonucleotide array designed specifically for the interrogation of copy number changes without the necessity to genotype SNPs. In addition, we have utilized a CNV detection algorithm that takes advantage of well established standardization methods [37,49,50] as well as the use of tree partitioning to segment the genome and delineate the CNV borders, a method that has been previously described for the identification of copy number changes using high density arrays [51] and is a powerful alternative to other segmentation algorithms [52-55]. We have further justified the use of a tree partitioning model coupled with a permutation test by extensive experimental validation of the CNV calls as well as the precision of the borders determined by the algorithm.

The single largest advantage of high density DNA oligonucleotide arrays is the vast amount of genetic information generated in a single experiment through the use of millions of independent probe sequences [56-58]. The increased value of higher density is evident based on the increased number of CNVs called in any pair wise comparison, and the ability to detect much smaller CNVs compared to other array based platforms [7]. For example, we identified 169 validated CNVs in one pair wise comparison (NA15510 vs NA10851) alone. This far outnumbers the list of CNVs discovered (using the same test and reference sample) by at least 5 other microarray based platforms (See Supplementary Table 1 in [59]) although is still less than the 241 alterations discovered by fosmid end sequencing of NA15510 [15]. Remarkably, in this one sample alone, more than 500 distinct copy number variations have been identified, and half of these have been experimentally validated. This underscores the point that any two human genomes may differ by tens of Megbases of DNA sequence due to structural variation alone.

One issue with CNV survey studies to date is the lack of overlap between variants identified using different platforms [59-61]. In addition, although the databases cataloguing all published CNV regions contain hundreds of Mbs of DNA, it is still unclear if a large proportion of these CNVs may in fact be false positives [59,62]. We have high confidence in the CNVs reported here since all have been experimentally validated or have been identified by multiple technological platforms.

The presence of non-polymorphic probes improves array performance by allowing more probes to be utilized, even in more complex regions of the genome, such as segmental duplication regions, which are often not accessible through standard SNP genotyping. Future whole genome association studies should utilize both SNPs and CN probes to maximize the information and content. While SNP detection has been widely used and tested, this is the first report of a non-polymorphic set of probes that can be evaluated for eventual inclusion onto an integrated array containing both polymorphic and non-polymorphic probes [47,61]. A subset of probes from the Nsp CN array has been empirically selected for maximum responsiveness and has been incorporated into the SNP 6.0 array [63]. This array is currently being used to assess structural variation in large sample sets. Finally, the Nsp CN arrays have been shown to be capable of detecting cancer causing aberrations with known pathological consequences [64]. Thus, this type of array could also be used for array-based karyotyping in lieu of more time consuming and expensive cytogenetic methods [65].

## Methods

### Array Design

The Nsp CN array contains 12,339,139 oligonucleotide probes tiled onto two arrays. Probes were selected to represent each of the 1,330,354 fragments between 200–1100 bp predicted to arise after digestion of human genomic DNA with the restriction enzyme NspI. All data presented is based on the human reference genome build 35 (May 2004 build). For all chromosomes, 8–10 PM (perfect match) probes were identified per fragment using a probe selection algorithm previously developed for high density 25-mer arrays [66]. Simple repeats and SNP sequences were avoided.

For background estimation, a pooled set of "antigenomic" probes were used which has been matched to each perfect match feature based on its GC content and which are not present elsewhere in the genome [42].

### Data Analysis

#### I. Preprocessing

##### 1. Probe Filtering

In order to extract the highest quality data from the Nsp-CN arrays, several filtering steps were implemented to remove adversely performing probes.

###### Probe filtering based on probe GC content, fragment length and GC content, and NspI restriction site characteristics

Several previous studies have suggested that the restriction fragment length and GC content as well as probe GC content have a strong effect on feature intensity [37,52,67]. Analysis of the relationship between Nsp-CN array probe intensity and its associated probe and fragment characteristics (data not shown) have led to the first set of filtering criteria: probes with less than 30% or greater than 60% GC content were removed as well as probes within restriction fragments greater than 1000 bp in length, <25% GC content, or > 60% GC content. In addition, probes residing in fragments in which the enzyme recognition site contains a SNP [68] were also filtered out.

###### Probe filtering based on number of genome hits

The xMAN (extreme Mapping of OligoNucleotides) algorithm was used to map all Nsp CN probes to the human genome [69]. Probes with more than two genomic hits were discarded due to reduced ability to respond to changes in target dosage.

After the above two filtering steps, the number of probes was reduced from 12,339,139 to 10,379,759 (84.12%), and the number of fragments were reduced from 1,330,354 to 1,245,607 (93.6%). The remaining set of filters was applied independently for each data set.

###### Filtering of high-intensity probes

Exploratory data analysis discovered that probes with the highest intensity on the arrays had very low dose response (Additional File 3), in part due to cross hybridization with multiple sites in the genome. For each set of samples being analyzed together, probes that were consistently in the top 10% intensity categories were filtered out.

###### Filtering of low-intensity probes: estimation of background effects

In order to identify probes that consistently failed to produce a signal above the background level, a sequence specific model was used to estimate the contribution of systematic noise to the probe signal intensity. Although overall probe GC content plays a crucial role in the estimation of background, recent studies have pointed out that position dependent sequence effects are also important [70-72]. Motivated by the sequence-specific model, the following multiple linear regression model was used to describe the background effect on the Nsp CN arrays:

$$\begin{array}{c} \log(Intensity_{i})=\alpha +\sum_{k\in \{A,C,G\}}\sum_{l=1}^{3}\beta_{k,l}P_{i,k}^{l}+\sum_{j=1}^{25}\sum_{k\in \{A,C,G\}}\sum_{l=1}^{3}\gamma_{k,l}j^{l}I_{ijk}\\ +\sum_{m=1}^{24}\sum_{n\in \{A\{A,C,G,T\},C\{A,C,G,T),G\{A,C,G,T),T\{A,C,G\}\}}\sum_{l=1}^{3}\delta_{n,l}m^{l}I_{imn}+\varepsilon_{i} \end{array}$$

where

• *Intensity*_*i *_is the probe intensity of probe *i*;

• *α *is the intercept of the regression;

• *j *= 1,...,25, representing the position along the probe *i*;

• *k *represents the base at position *j*;

• *P*_*i*,*k *_is the percentage of nucleotides A, C, G in the probe *i*;

• *β*_*k*,*l *_is the effect of nucleotide percentage (A, C, or G) in the probe, for a fixed base nucleotide *k*, the effect is modeled as a polynomial of degree 3;

• *I*_*ijk *_is an indicator function such that it is 1 when the *j*th position is base *k *in probe *i*, and it is 0 otherwise;

• *γ*_*k*,*l *_is the effect of base *k *in position *j*, the effect is modeled as a polynomial of degree 3;

• *m *= 1,2,...,24, representing the di-nucleotide position along the probe *i*;

• *n *is the set of di-nucleotide nearest neighbor compositions such as 'AA', 'AC', 'GT' etc;

• *I*_*imn *_is an indicator function such that it is 1 when the *m*th position is di-nucleotide *n *in probe *i*, and it is 0 otherwise;

• *δ*_*n*,*l *_is the effect of di-nucleotide in position *m*, the effect is modeled as a polynomial of degree 3;

• *ε*_*i *_is the error-term.

Log intensities of all 33,886 anti-genomic probes were fitted to estimate parameters using least squares. Each array was fitted separately and a total of 64 parameters were estimated for each array. These parameters were used to calculate the background-adjusted intensities for all interrogation probes on the array, and the value of zero was set as the threshold to determine whether signal was greater than background. For each set of samples being analyzed together, probes that exhibited a consistent signal lower than background were filtered out.

###### Probe filtering based on number of probes within a fragment (probe set)

The last probe filtering step removed probes where only a single probe remained for a given fragment (due to filtering from previous steps). Thus, every fragment is represented by at least two probes that have passed all filtering criteria.

##### 2. Probe Standardization

Inspired by previous studies demonstrating that probe intensities are affected by fragment length, fragment GC content, probe GC content, nucleotide locations on the probe, and recognition site sequence of restriction enzyme, optical background adjusted probe intensities were fitted to a multiple linear regression model [37,70-72]. The AIC stepwise auto-selection procedure was used to identify the best model. The starting model has a 10 degree polynomial for each variable. A cubic term was used with most of the variables and the subset of selected variables can be slightly different from sample to sample. The following multiple linear regression model was used to fit the data:

$$\begin{array}{c} \log(\text{adjusted}PM_{i})=\alpha +\sum_{k\in \{A,C,G\}}\sum_{l=1}^{3}\beta_{k,l}P_{i,k}^{l}+\sum_{j=1}^{25}\sum_{k\in \{A,C,G\}}\sum_{l=1}^{3}\gamma_{k,l}j^{l}I_{ijk}\\ +\sum_{m=1}^{24}\sum_{n\in \{A\{A,C,G,T\},C\{A,C,G,T),G\{A,C,G,T),T\{A,C,G\}\}}\sum_{l=1}^{3}\delta_{n,l}m^{l}I_{imn}\\ +\sum_{o\in \{A,C,G\}}\sum_{l=1}^{3}\eta_{o,l}F_{i,o}^{l}+\sum_{l=1}^{3}\lambda_{i,l}L^{l}+\zeta_{i}I_{ic=C}+\varepsilon_{i} \end{array}$$

where

• adjusted *PM*_*i *_is the optical background adjusted probe intensity of probe *i*; for each array, the minimum intensity from all interrogation probes is first identified and this number minus 1 is regarded as the optical background intensity and it is subtracted from all probe intensities;

• *α*, *j*, *i, k, P*_*i*,*k*_, *β*_*k*,*l*_, *I*_*ijk*_, *γ*_*k*,*l*_, *m*, *n*, *I*_*imn*_, *δ*_*n*,*l*_, *ε*_*i *_have the same meaning as in formula (1);

• *F*_*i*,*o *_is the percentage of nucleotide A, C, or G in the fragment on which probe *i *resides;

• *η*_*o*,*l *_is the effect of A, C, or G percentage in the fragment, for a fixed base nucleotide *o*, the effect is modeled as a polynomial of degree 3;

• *L *is the length of the fragment which corresponds to probe *i*;

• *λ*_*i*,*l *_is the effect of fragment length, the effect is modeled as a polynomial of degree 3;

• *I*_*ic *_is an indicator function such that it is 1 when the nucleotide at the 3' restriction cutting site is C and it is 0 otherwise;

• *ζ*_*i *_is the effect of nucleotide at the 3' restriction cutting site for the fragment on which probe *i *resides;

There are total of 77 parameters in this model consisting of 1 α, 9 β, 45 γ, 9 δ, 9 η, 3 λ and 1 ζ. 100,000 autosomal probes were randomly selected from probes which were kept after filtering steps for each array. Optical background adjusted intensities from these 100,000 probes were used to fit the model to estimate the model parameters for each array. Using these estimated parameters, residual intensities for all probes were predicted and these standardized intensities were used in subsequent steps.

##### 3. Probe Set Summarization

After filtering and standardization, probes residing on the same Nsp I restriction fragment (i.e. the probe set) were summarized to a single value using RMA, a median polish based method developed previously for RNA expression studies to account for feature effects due to probe composition [73]. The effect of RMA was evaluated using the 1X–5X DNA samples, where the linear correlation coefficient and the regression slope improved significantly (Additional File 5).

###### Pair-wise CNV Detection

CNV detection was implemented on a pair-wise basis by comparing a single test sample with a single reference sample. In this study, we only concentrate on discovering CNVs from autosomal chromosomes. Immuglobulin genes (Ig) were removed from the analysis. These regions include IgK at 2p11, IgL at 22q11, and IgH at 14q32[74].

#### II. Genome Segmentation

##### 1. Calculating log of intensity ratio

After the RMA summarization step, each probe set is represented by one single value. Subsequently, log intensities of the reference sample were subtracted from the test sample to obtain the log intensity ratio.

##### 2. Local correction

A local correction step was used to remove outlier fragments based on the premise that a typical CNV region should span more than one *NspI *fragment, and neighboring fragments within a CNV should have a similar log intensity ratio. First, all significant fragments from each chromosome were identified as fragments whose log intensity ratio is 3 times higher than the chromosome specific standard deviation of the log intensity ratio. A single non-significant fragment located between two significant fragments was ignored for subsequent analysis as long as the significant fragments were in the same direction (either positive log intensity ratios or both negative log intensity ratios). Furthermore, if the two significant fragments were very close in distance (<1 kb), all non-significant fragments located between them were removed. In addition, a single significant point was removed if neighboring points, defined as the nearest upstream or downstream fragment within 100 kb, or any fragment within 1 kb, did not show a log intensity ratio greater than 2 times the standard deviation of log intensity ratios. For a typical pair-wise comparison, 0.8%–0.9% of the fragments were filtered out in this step.

##### 3. Kernel smoothing and regression tree partitioning to identify CNV regions

To make array data more comparable across different data sets, the local-corrected intensities were first scaled to a mean of zero by subtracting the mean log intensity ratio for all autosomal fragments. Next, to improve the signal to noise of the adjusted log intensity ratio data, kernel smoothing was applied with a Gaussain kernel and a 10 kb bandwidth. Finally, in order to identify putative CNV regions, the smoothed log intensity ratios were fitted to a regression tree model as described previously [51,75]. The end result is the partitioning of the genome into consecutive genomic regions. A single measurement is derived from each region which is the mean log intensity ratio based on all fragments that are within the region.

The optimal value for the threshold complexity parameter (cp), was empirically determined using a test sample, NA15510 and a reference sample, NA10851. This parameter controls the complexity of the partitioning of the regression tree. We tested a range of cp values, from 0.0001 (used in our previous study [51]) to 0.001 in a step of 0.0001. The two major metrics used to evaluate this parameter were 1) how well the final CNV list overlapped with validated/reported known CNV regions in sample NA15510 [7,15], and 2) whether regions either known to undergo somatic rearrangement (such as the Ig loci) or harbor previously identified CNVs are split into several smaller regions. This cp parameter was finally set to 0.0004, indicating that splits which do not increase the overall R-squared value by 0.04% were not tested. In the process of building the regression tree, the "minsplit" parameter was set to 3. When a genomic region contains 3 or less fragments, the tree building procedure was halted. In the tree-pruning phase of the algorithm, 10 fold cross-validation and the 1-SE (standard deviation) rule were used to decide the size and the complexity of the final tree model [51,75].

#### III. CNV Identification

##### 1. Permutation approach to define CNV significance thresholds

To determine significance thresholds for defining CNV regions in pair-wise comparisons, we used a permutation test after the local correction and mean ratio adjustment. The physical locations of the fragments were randomly permuted 500 times and the permuted data was subjected to the same kernel smoothing and regression tree partitioning procedures with the same algorithmic parameters as described above. A unique threshold was defined for each size group based on the false discovery rate (FDR).

Genome partitioning results from the permutation runs were parsed into 19 size groups containing 2, 3, 4, 5, 6, 7, 8, 9, 10, 11–15, 16–20, 21–30, 31–40, 41–50, 51–60, 61–70, 71–80, 81–90, and 91–100 fragments to get size specific null distributions of the log intensity ratios. A unique threshold was defined for each size group based on the false discovery rate (FDR) [76,77] with even partitioning of the FDR among all the size groups. The following formula was used to determine the significance threshold for each size group:

$$I_{ij}=\frac{fdr}{N_{g}*N_{a}}*\frac{N_{pij}}{N_{cij}}$$

where

• *I*_*ij *_is the index for retrieving the significance threshold for size group *i *on array *j *of the Nsp-CN array set, *j *= 1,2;

• *fdr *is the pre-specified maximal false discovery rate for the whole Nsp-CN array set;

• *N*_*g *_is the total number of size groups;

• *N*_*a *_is the number of arrays in the array set, *N*_*a *_= 2 for Nsp CN array set;

• *N*_*pij *_is the number of genomic regions in the size group *i*, based on results summarized from all the permutation runs of array *j*;

• *N*_*cij *_is the number of genomic regions in the size group *i*, based on results from tree partitioning of the test sample's genome on array *j*;

Once the *I*_*ij *_was computed, *I*_*ij *_+ 1 was the index used to retrieve significance thresholds for size group *i *on array *j*. Thresholds for amplifications and deletions were computed separately. Significant regions from the partitioned test sample were identified using these log intensity ratio thresholds. For putative CNV regions containing more than 100 fragments, which were not considered directly in the permutation test, we used the threshold derived from the 91–100 fragment group and required the log intensity ratio to be greater than 3 times the standard deviation of raw, unsmoothed autosomal log intensity ratios.

The optimal number of falsely-detected CNVs for our test sample was identified as eight (after testing values between 1 and 10) using the following criteria: 1) overlap of generated CNV regions with reported CNVs in the literature and 2) consistency with QPCR validation. This number corresponds to a FDR (False Detection Rate) of ~5% since there are ~160 CNVs detected in each pair-wise comparison (8/160).

##### 2. Additional criteria for determining the final CNV regions

To generate the final list of CNV regions, the following additional steps were taken:

1) Only putative CNV regions with average log intensity ratios greater than 4 times the standard deviation of kernel smoothed, autosomal log intensity ratios were retained.

2) Adjacent significant regions were merged to form one larger CNV region and the log intensity ratio of the newly merged region was averaged.

3) Only CNVs containing more than one significant fragment were retained. Significance was based on having a raw log intensity ratio at least 3 times more significant than the standard deviation of raw, un-smoothed autosomal log intensity ratios.

### Target preparation and hybridization to arrays

DNA from cell lines was purchased from the Coriell Institute for Medical Research (Camden, NJ). The DNA samples containing different numbers of X chromosomes (1X to 5X sample set) are NA10851, NA15510, NA04626, NA01416 and NA06061. The sample used for much of the parameter tuning and CNV identification was the test sample, NA15510. Additional samples include two HapMap trios (NA10831, NA12155, NA12156, NA10846, NA12144, NA12145). In all cases a normal male reference sample, NA10851, was used for comparison.

For target preparation of the DNA, we used the whole genome sampling assay (WGSA) as described by the manufacturer for the Nsp250K SNP genotyping array [63]. Briefly, 250 ng of DNA is digested with NspI, adapter-ligated, and PCR amplified using a single primer homologous to the adapter. After purification, 90 ug of fragmented and labeled target is hybridized onto the array.

For data quality assessment, genotype calls were generated from 250 SNPs using the DM (Dynamic Modeling) calling algorithm with cutoff p-value 0.26 [78]. Any arrays giving rise to a call rate of less than 85% were redone.

### QPCR validation of CNV regions

Quantitative PCR using the ABI 7500 Sequence Detection System was used to independently validate CNVs detected by our algorithm as described previously [37]. At least four replicate reactions for novel CNVs were run for each primer pair and the comparative ΔΔC_T _method (User Bulletin #2; Applied Biosystems) was used to calculate the fold change at each locus between the test and reference samples. In addition, a t-test p-value based on the ΔCt values was used to determine the statistical significance of the result. The thresholds for determining whether an amplicon was validated or not were set using results from seven independent X chromosome amplicons that were each analyzed using the 1X to 5X DNA samples (Additional File 7). The 1X, 3X, 4X and 5X DNA samples were compared to the normal female 2X sample for each of the seven amplicons for a total of 28 measurements (4 comparative measurements per amplicon × 7 amplicons). All results that showed a fold change less than 0.8 or greater than 1.25 as well as a p-value < 0.01 were considered to be significant. Using these thresholds, there were 24 of the 28 comparisons that reached significance. Of the four measurements that did not meet significance, one (Chr X_Amplicon 2) is a known copy number variant between NA15510 (2X sample) and NA18501 (1X sample) and thus this did not pass the fold change threshold. The remaining three measurements all passed the fold change threshold but did not pass the p-value cut-off. For ambiguous results, the QPCR was repeated and often new primer pairs were designed as shown in the Additional File 6. Of the 96 QPCR-validated CNVs, 18 were tested with a single amplicon and 76 were tested with at least two independent amplicons. Also, for CNVs that failed QPCR validation, 23 out of 25 were tested with two or more amplicons. Any one primer pair displaying significance was considered evidence of CNV validation. Some novel CNVs reside in regions of segmental duplication that preclude the identification of QPCR primer pairs that generate a single unique amplicon. Thus independent validation of these CNVs is technically challenging, leading to possible false negative results.

### Data Release

The raw data from this study are posted at the Gene Expression Omnibus with accession number GSE9053 [79].

## Authors' contributions

FS and JH developed and implemented the algorithm; all codes are written in R version 2.2.0 and perl5. AK and SM were involved in algorithm discussions. VT, WC, JZ, GL, KF, KJ, and MS were involved in the array data generation and independent verification using PCR molecular biology approaches. GL was involved in bioinformatics analysis related to the array design. FS, KRF, and MHS wrote the manuscript and all authors read and approved the final manuscript.

## Supplementary Material

Additional file 1 Dose response plots of a representative 1X–5X data set. Panels a-d show the scatter plots of standardized natural log intensity of the 1X, 3X, 4X, and 5X samples relative to the 2X sample. Here, standardization refers to the following data transformation: standardized intensity of chromosome X probe = (intensity of chromosome X probe-mean intensity of the autosomal probes)/standard deviation of the intensity of autosomal probes. Red dots represent randomly selected chromosome X probes and black dots represent randomly selected autosomal probes. The blues lines are the Y = X lines. Panel e shows the relationship between the natural log-transformed intensity and the natural log-transformed copy number. Natural log-transformed mean intensity of all chromosome X probes from the 1X–5X samples are plotted on the Y-axis and natural log-transformed copy number are plotted on the X-axis. The blue line is the linear regression line using the natural log-transformed mean intensity as response and natural log-transformed copy number as predictors. Click here for file

Additional file 2 Dose response of probes deteriorates as the number of genomic hits increases. Panel a shows the frequency distribution of genomic matches for a set of 80,000 randomly selected chromosome X probes. Panels b-c are box-plots showing the distribution of linear correlation coefficient and regression slope grouped by the number of genomic hits of a set of 80000 randomly selected chromosome X probes. Panel d shows chromosome X hits frequency distribution of the same set of randomly selected 80000 chromosome X probes. Panels e-f are box-plots showing the distribution of linear correlation coefficient and regression slope grouped by the number of chromosome X hits of this set of 80,000 randomly selected chromosome X probes. Natural log-transformed normalized (as described in Methods) intensity of chromosome X probes of a representative set of 1X–5X samples and natural log-transformed copy number were used to calculate linear correlation coefficient and regression slope for each probe. Click here for file

Additional file 3 A 2-dimensional histogram showing the distribution of regression slope along with the distribution of natural log-transformed intensity. Natural log-transformed normalized (as described in Methods) intensity of 80,000 randomly selected chromosome X probes of a representative set of 1X–5X samples and natural log-transformed copy number were used to calculate the regression slope. The black vertical line denotes the maximum log intensity ratio and the green vertical line denotes the top 8% log intensity, above which there are few probes with high regression slopes. The top 10% intensity is used as the cut-off threshold in the probe filtering process. Click here for file

Additional file 4 Number of remaining probes and fragments following probe filtering for 3 replicates of 1X–5X samples. The data indicates the number of probes and fragments that have been retained after probe filtering for 3 replicates of the 1X–5X DNA samples. Click here for file

Additional file 5 Dose response of probes improves after probe filtering and RMA procedure. Natural log-transformed normalized (as described in Methods) intensity of 80,000 randomly selected chromosome X probes of a representative set of 1X–5X DNA samples and natural log-transformed copy number were used to calculate linear correlation coefficient and regression slope for all probes(blue bars), natural log-transformed normalized intensity of post-filtering 64,035 of the 80,000 randomly selected chromosome X probes and natural log-transformed copy number were used to calculate linear correlation coefficient and regression slope for the filtered probes(grey bars), and natural log-transformed post-RMA chromosome X probe set intensity and natural log-transformed copy number were used to calculate linear correlation coefficient and regression slope for the fragments (red bars). Click here for file

Additional file 6 List of QPCR data and CNV coordinates. Table A represents the coordinates of CNVs in NA15510 vs. NA10851. Table B summarizes QPCR results for NA15510 vs. NA10851. Table C represents QPCR results for the CNV border analysis. Table D represents QPCR results for Mendelian inheritance (MI) errors. Table E lists counts of CNVs in HapMap trio samples NA10846-NA12144-NA12125 and NA10831-NA12155-NA12156. Click here for file

Additional file 7 Chromosome X QPCR Analysis. The data represents QPCR analysis of seven independent X chromosome amplicons that were each analyzed using the 1X to 5X DNA samples. Click here for file
