# Supplementary figures and images for: Improved detection of global copy number variation using high density, non-polymorphic oligonucleotide probes
Source: BMC Genet. 2008 Mar 28;9:27. doi: 10.1186/1471-2156-9-27 (PMC2374799; doi:10.1186/1471-2156-9-27)

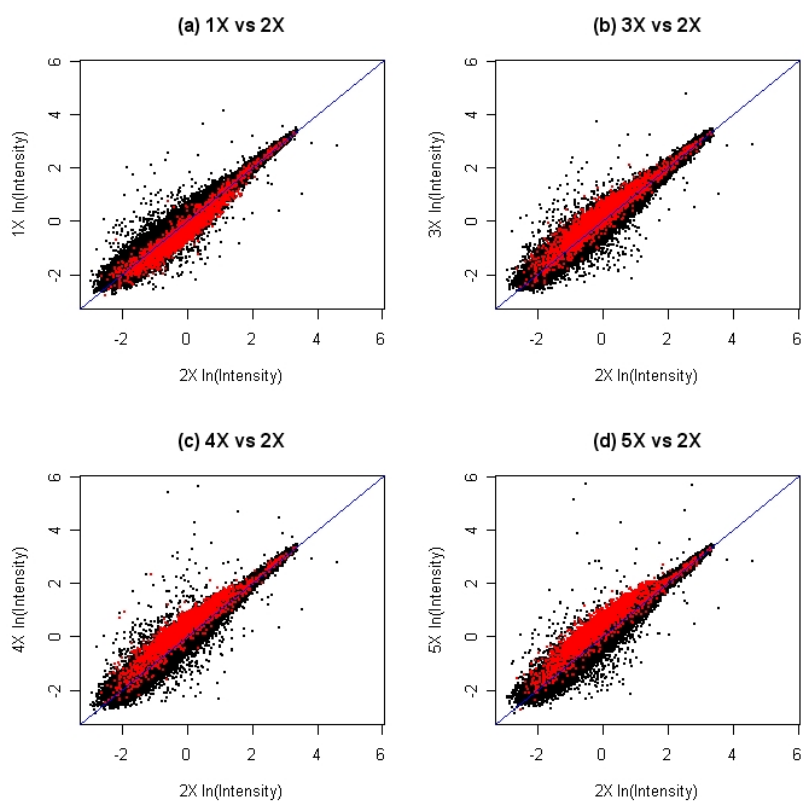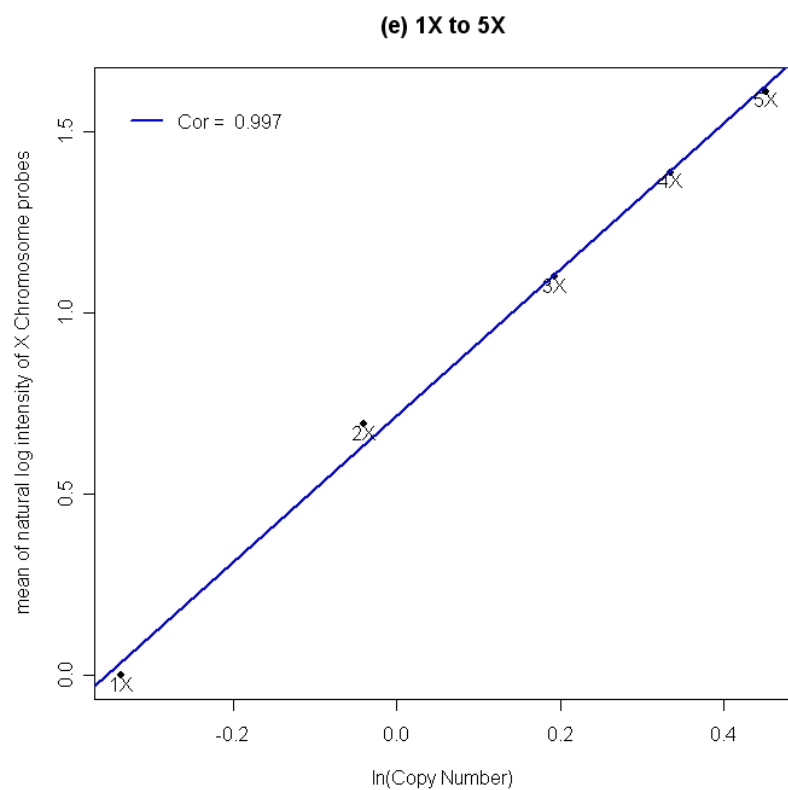

Supplement: Additional file 1 — Dose response plots of a representative 1X–5X data set. Panels a-d show the scatter plots of standardized natural log intensity of the 1X, 3X, 4X, and 5X samples relative to the 2X sample. Here, standardization refers to the following data transformation: standardized intensity of chromosome X probe = (intensity of chromosome X probe-mean intensity of the autosomal probes)/standard deviation of the intensity of autosomal probes. Red dots represent randomly selected chromosome X probes and black dots represent randomly selected autosomal probes. The blues lines are the Y = X lines. Panel e shows the relationship between the natural log-transformed intensity and the natural log-transformed copy number. Natural log-transformed mean intensity of all chromosome X probes from the 1X–5X samples are plotted on the Y-axis and natural log-transformed copy number are plotted on the X-axis. The blue line is the linear regression line using the natural log-transformed mean intensity as response and natural log-transformed copy number as predictors. [file 1471-2156-9-27-S1.pdf]

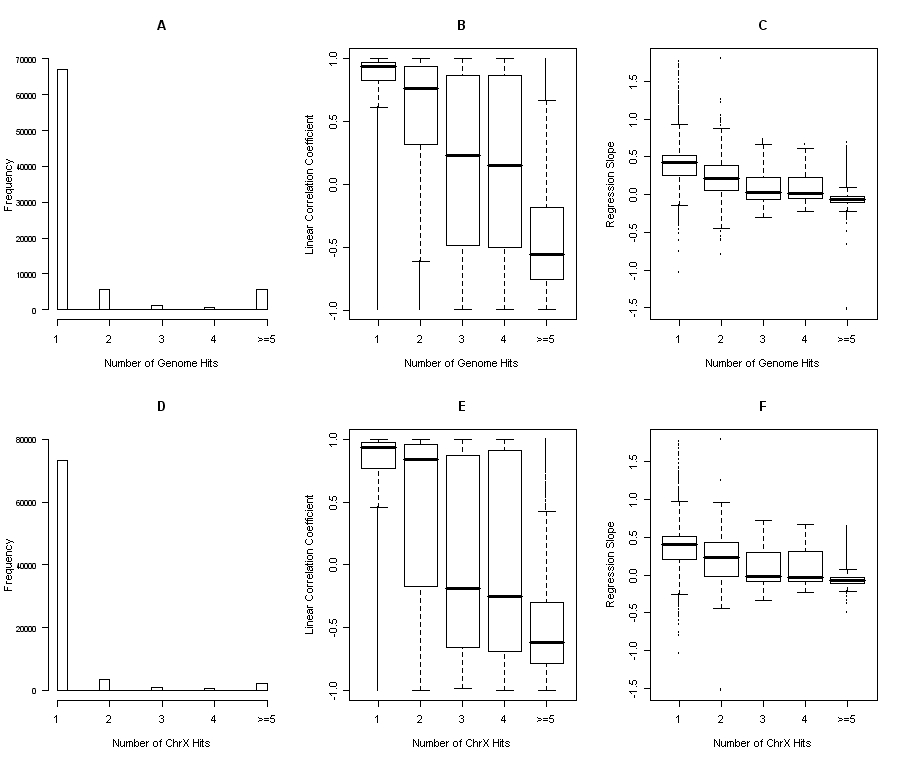

Supplement: Additional file 2 — Dose response of probes deteriorates as the number of genomic hits increases. Panel a shows the frequency distribution of genomic matches for a set of 80,000 randomly selected chromosome X probes. Panels b-c are box-plots showing the distribution of linear correlation coefficient and regression slope grouped by the number of genomic hits of a set of 80000 randomly selected chromosome X probes. Panel d shows chromosome X hits frequency distribution of the same set of randomly selected 80000 chromosome X probes. Panels e-f are box-plots showing the distribution of linear correlation coefficient and regression slope grouped by the number of chromosome X hits of this set of 80,000 randomly selected chromosome X probes. Natural log-transformed normalized (as described in Methods) intensity of chromosome X probes of a representative set of 1X–5X samples and natural log-transformed copy number were used to calculate linear correlation coefficient and regression slope for each probe. [file 1471-2156-9-27-S2.jpeg]

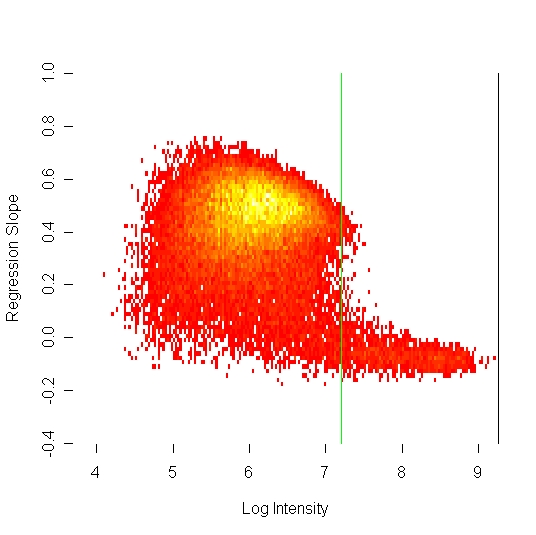

Supplement: Additional file 3 — A 2-dimensional histogram showing the distribution of regression slope along with the distribution of natural log-transformed intensity. Natural log-transformed normalized (as described in Methods) intensity of 80,000 randomly selected chromosome X probes of a representative set of 1X–5X samples and natural log-transformed copy number were used to calculate the regression slope. The black vertical line denotes the maximum log intensity ratio and the green vertical line denotes the top 8% log intensity, above which there are few probes with high regression slopes. The top 10% intensity is used as the cut-off threshold in the probe filtering process. [file 1471-2156-9-27-S3.jpeg]

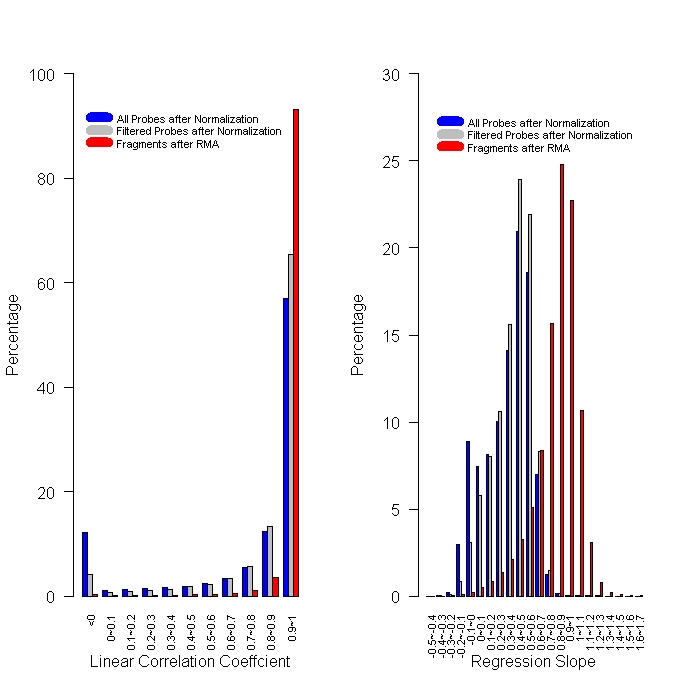

Supplement: Additional file 5 — Dose response of probes improves after probe filtering and RMA procedure. Natural log-transformed normalized (as described in Methods) intensity of 80,000 randomly selected chromosome X probes of a representative set of 1X–5X DNA samples and natural log-transformed copy number were used to calculate linear correlation coefficient and regression slope for all probes(blue bars), natural log-transformed normalized intensity of post-filtering 64,035 of the 80,000 randomly selected chromosome X probes and natural log-transformed copy number were used to calculate linear correlation coefficient and regression slope for the filtered probes(grey bars), and natural log-transformed post-RMA chromosome X probe set intensity and natural log-transformed copy number were used to calculate linear correlation coefficient and regression slope for the fragments (red bars). [file 1471-2156-9-27-S5.jpeg]
